# Nano-pulse stimulation (NPS) ablate tumors and inhibit lung metastasis on both canine spontaneous osteosarcoma and murine transplanted hepatocellular carcinoma with high metastatic potential

**DOI:** 10.18632/oncotarget.17178

**Published:** 2017-04-18

**Authors:** Xinhua Chen, Yiling Chen, Jianwen Jiang, Liming Wu, Shengyong Yin, Xudong Miao, Robert J. Swanson, Shusen Zheng

**Affiliations:** ^1^ Collaborative Innovation Center for Diagnosis and Treatment of Infectious Diseases, Key Laboratory of Combined Multi-Organ Transplantation, Ministry of Public Health, Department of Hepatobiliary and Pancreatic Surgery, The First Affiliated Hospital, Zhejiang University, Zhejiang, 310003, China; ^2^ The Department of Anatomy, Tianjin Medical University, Tianjin, 300070, China; ^3^ The Department of Orthopedics, The Second Affiliated Hospital, Zhejiang University, Zhejiang, 310003, China; ^4^ Anatomical Sciences Department, College of Osteopathic Medicine, Liberty University, Lynchburg, VA, 24515, USA

**Keywords:** nano-pulse stimulation (NPS), nanosecond pulsed electric field (nsPEF), non-thermal, ablation, palliative therapy

## Abstract

**Background:**

Nanosecond pulsed electric field (nsPEF), which is also termed as nano-pulse stimulation (NPS), has the potential of stimulating immune responses toward cancer cells. The current study investigates its local and systemic antitumor efficacy *in vivo* in late stage tumors with lung metastasis.

**Method:**

The 12 canines with spontaneous osteosarcomas and 12 nude mice transplanted with human hepatocellular carcinoma were divided randomly and were given NPS treatment, surgery or no treatment control. Nanosecond pulsed electric field was delivered with puncture electrodes at 40 kV/cm with 500 pulses at 1 Hz. The survival time, tumor volume, serum alkaline phosphatase (ALP), joint capsule damage and lung metastasis were followed up. The efficacy was compared with control.

**Results:**

Nanosecond pulsed electric field reduced primary tumor volume and extended the survival significantly compared to the control group (*P*<0.05). Inhibition of serum alkaline phosphatase and lung metastasis without joint deformity or thermal damage were also observed.

**Conclusion:**

Locally applied nanosecond pulsed electric field is a novel non-thermal ablation method. It can ablate the primary tumor and decrease lung metastasis as a palliative therapy for late stage tumor.

## INTRODUCTION

Nanosecond pulsed electric field (nsPEF) ablation originated from high voltage power technology and it showed potential in loco-regional tumor ablation [1]. NsPEF ablates tumors with ultra-short pulses by altering electrical conductivity and permeability of the tumor cell membrane, causing cell apoptosis [2, 3]. Recently its potential in stimulating immune reaction was also reported [4, 5, 6]. Due to its potential to induce immune responses toward cancer cells, it is also termed as nano-pulse stimulation (NPS) [7].

Previous studies showed nanosecond pulsed electric field can ablate different solid tumors such as skin tumors and hepatocellular carcinomas [1–7], but the tumors originating from bone have never been studied. Electric fields with the correct parameters can enhance bone reforming which might be helpful for bone tumors with pathological fracture. The late stage tumor with lung metastasis is another challenge in oncogene practice. We thus investigated the antitumor effect of nsPEFs in two different *in-vivo* tumor models with lung metastasis.

## RESULTS

### The clinical features of tumor-bearing animal models

A total of 12 dogs with spontaneous osteosarcoma and 12 nude mice transplanted with high metastatic human HCC cell line HCCLM3 were enrolled in the study [8–10]. They had no prior treatment regimens including surgery, radiation therapy, chemotherapy, or a combination of these treatments. Details of age, weight, and staging were listed in Table 1 and Table 2 respectively.

**Table 1:** Summary of the clinical characters of canine spontaneous osteosarcoma

	nsPEF (n=4)	control (n=5)	amputation (n=3)
**Weight**	20.2±3.8	17.6±2.1*	21.5±4.3
**Complications**	1/5	4/4	1/3
**Staging**	stage III	stage III	stage III
**Hospitalization (day)**	0.5±0.5	/	3.5±1.5

Canine osteosarcoma staging adapts the TNMG" system (T-tumor, N-node, M-metastasis, G-grade). Stage I includes low-grade tumors (G1) without evidence of metastasis; stage II includes high-grade tumors (G2) without metastasis; and stage III includes dogs with metastatic disease.

**Table 2:** Summary of the clinical characters of murine transplanted hepatocellular carcinoma with high metastatic potential

	nsPEF (n=6)	control (n=6)
**Weight (g)**	20.2±6.4	21.3±4.2*
**Complications**	0/6	0/6
**Primary tumor-T1**	6/6	6/6
**Regional lymph nodes -Nx**	6/6	6/6
**Lung metastasis**	6/6	6/6
**Staging**	stage IVB	stage IVB

The murine transplanted HCC staging adapts The American Joint Committee on Cancer (AJCC) tumor/node/metastasis (TNM) classification. The 12 mice in this study were all in Stage IVB (Any T, Any N, M1). Note that these are based on the AJCC Cancer Staging Manual, 7th Edition (2010). Although the 8th edition has been published, implementation of the new system has been delayed until January 1, 2018. The AJCC advises that, “All newly diagnosed cases through December 31st 2017 should be staged with the 7th edition.”. Note: T1 refers to solitary tumor without vascular invasion; Nx reefers to regional lymph nodes cannot be assessed.

### Procedure feasibility

**Figure 1:**
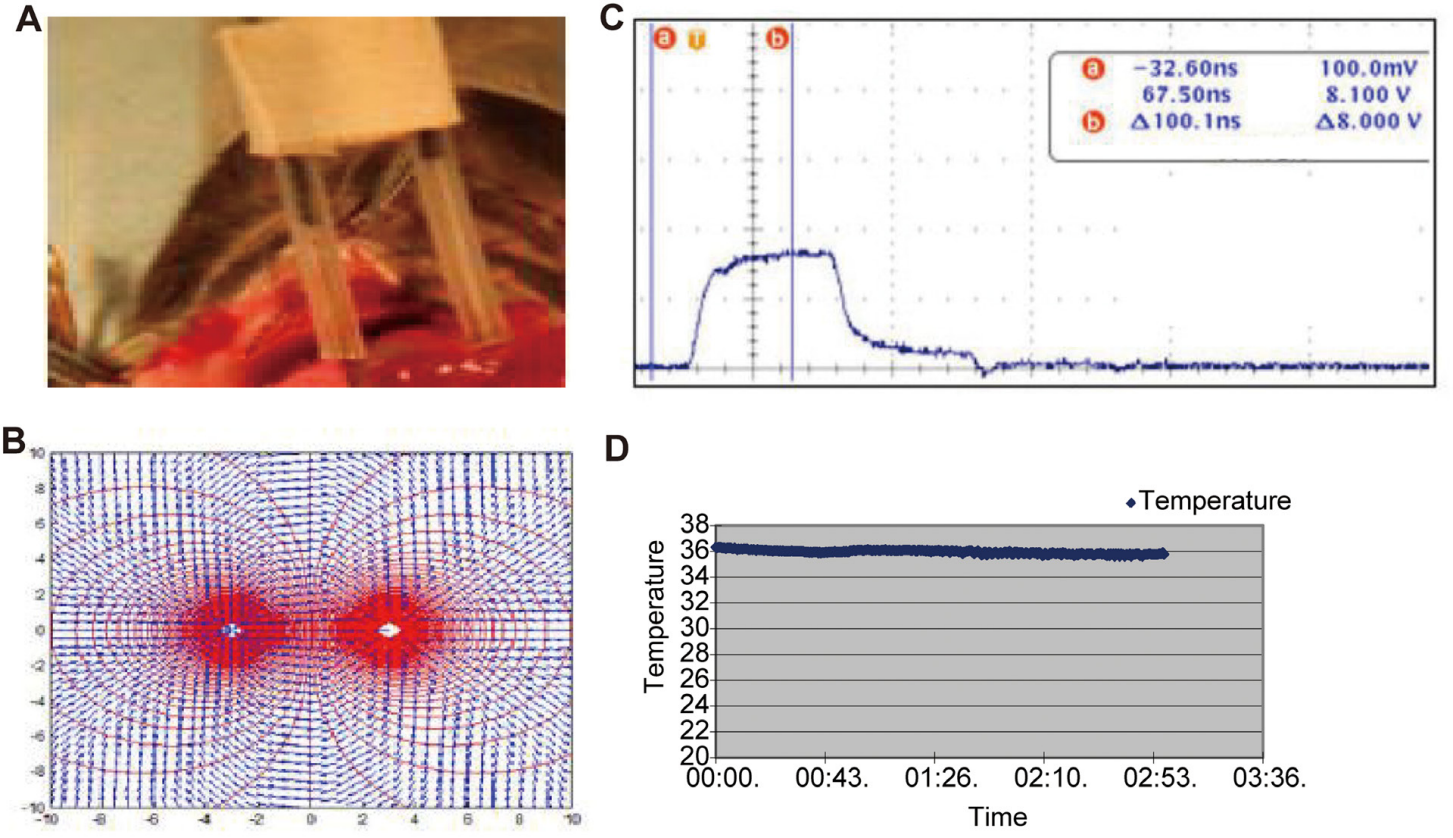
Pulse delivery assembly and nsPEF treatment A pair of needle electrodes were used to deliver electric field into the tumor. They were electrically insulated 1cm from tip **(Figure 1A)**. The distance in between was 2cm. The electric field distribution was not uniform and formed an oval ablation zone **(Figure 1B)**. The machine generated the highest 40 kV/cm-electric field in the center **(Figure 1C)**. The tumor temperature during treatment was lower than 40°C **(Figure 1D)**.

The percutaneous ablation was proved to be feasible in tumor-bearing dogs and mice. The pulsed electrical field power was delivered with 100ns-pulses by the pair needle electrodes (Figure 1). The median ablation time on dogs was 25 minutes (range 25–65 minutes). The average amputation surgery time was 45 minutes per dog (range 20-90 minutes). The median hospitalization time of nsPEF ablation group (0.5±0.5 day) was less than surgery group (3.5±1.5 days).

### Safety during ablation

Electrocardiogram found no cardiovascular events such as supraventricular tachycardia, atrial fibrillation pneumothorax, or lower limb thrombosis. The vital signs were stable during ablation. The temperature does not exceed 40°C between the electrodes during ablation (Figure 1), thus hyperthermic damage on the adjacent tissue was avoided.

### Complication observation in dogs post ablation

Death and adverse events were retrospectively reviewed and assessed. The mortality was 0% in both surgery and nsPEF groups. Complications in the ablation group included one case of hemorrhage. Complications in the surgery group included 1 case of wound infection and bleeding in the needle track. The control group without any treatment had several end-stage tumor-related complications including two cases of dyspnoea (caused by lung metastasis), four cases of cachexia (caused by tumor nutrition consumption), one case of difficulty walking and seizures (caused by paraneoplastic syndrome).

### Survival study on dogs

**Figure 2:**
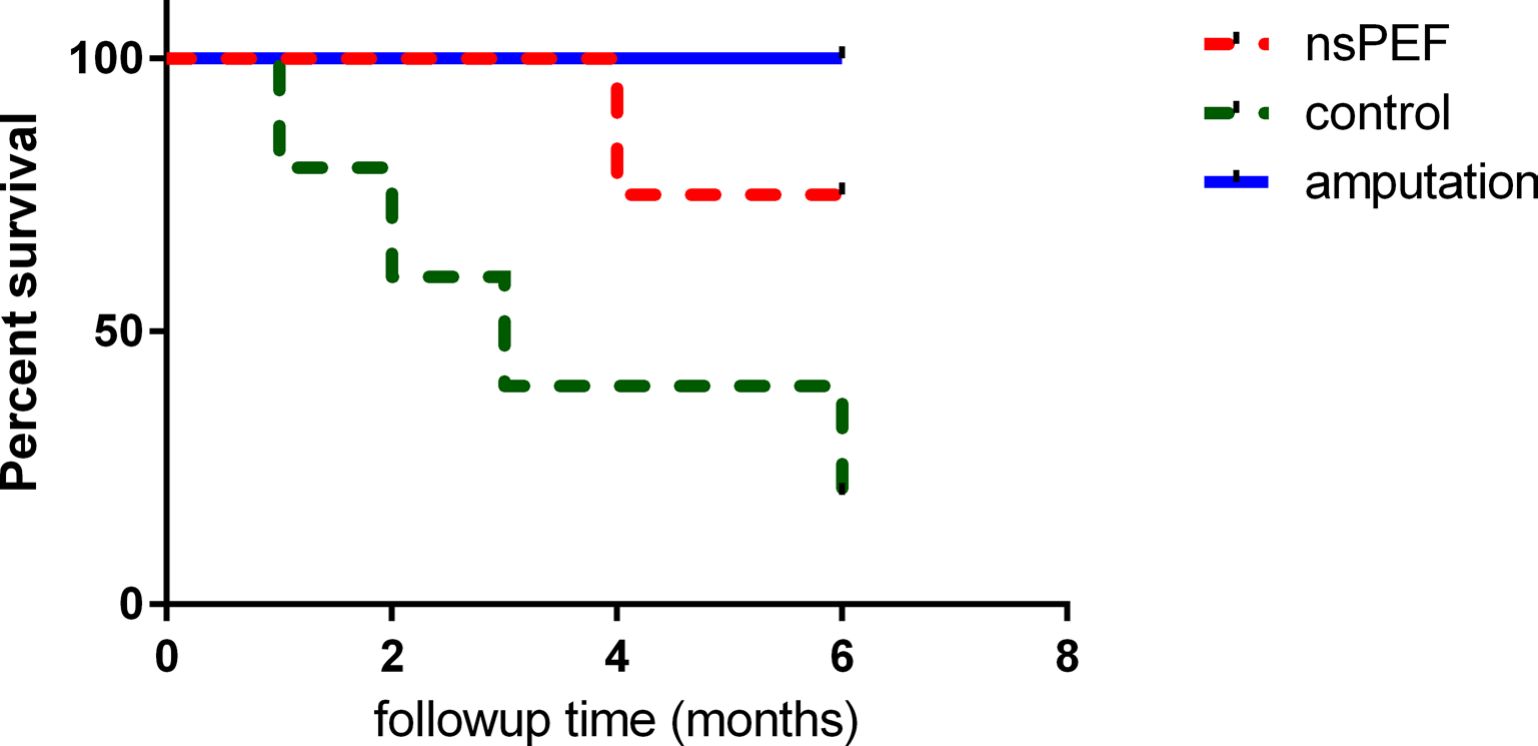
Survival study in canine spontaneous osteosarcoma After different treatments, the dogs were followed up for 6 months. Kaplan-Meier curves with log-rank tests were performed to investigate the differences of survival rate. Both surgery group and nsPEF ablation group survived significantly longer than control (P< 0.05).

The dogs were followed up to 185 days (Figure 2). Amputation provided a longer survival in the surgery group (3/3). NsPEF ablation helped 3 dogs survive 6 months (3/4). In the control group without any treatment 4 dogs died in 6 months. Log-rank (Mantel-Cox) test indicated nsPEF increased the survival compared to the control group (P <0.05).

### NsPEF inhibit primary tumor growth *in vivo*

**Figure 3:**
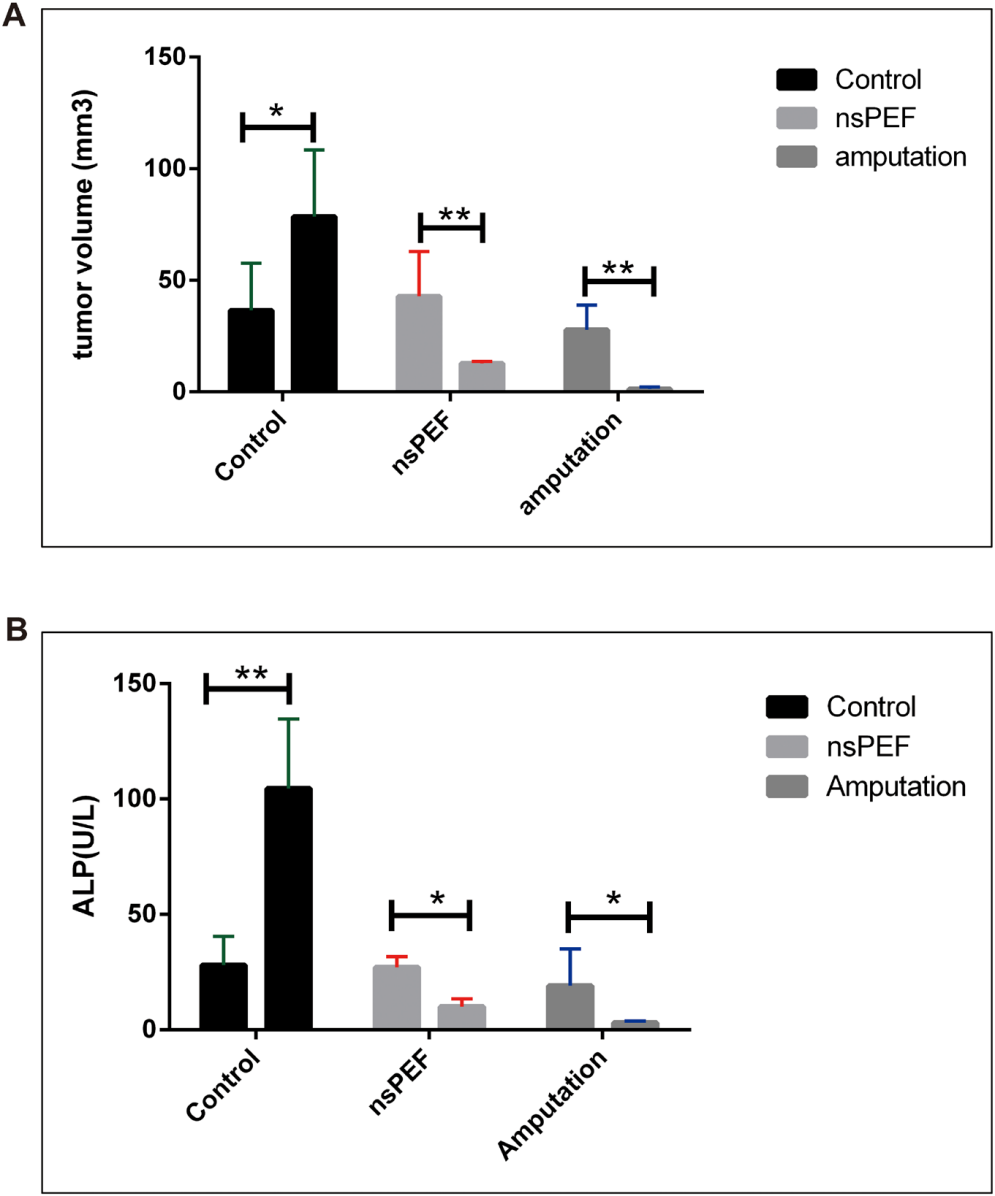
Tumor volume and serum alkaline phosphatase in canine spontaneous osteosarcoma After different treatments, the dogs were followed up for 6 months. The nsPEF ablation reduced the tumor size compared with the control group (P<0.05) **(Figure 3A)**. The alkaline phosphatase fell in surgery (P<0.001) and nsPEF ablation group (P<0.05) vs control group **(Figure 3B)**.

**Figure 4:**
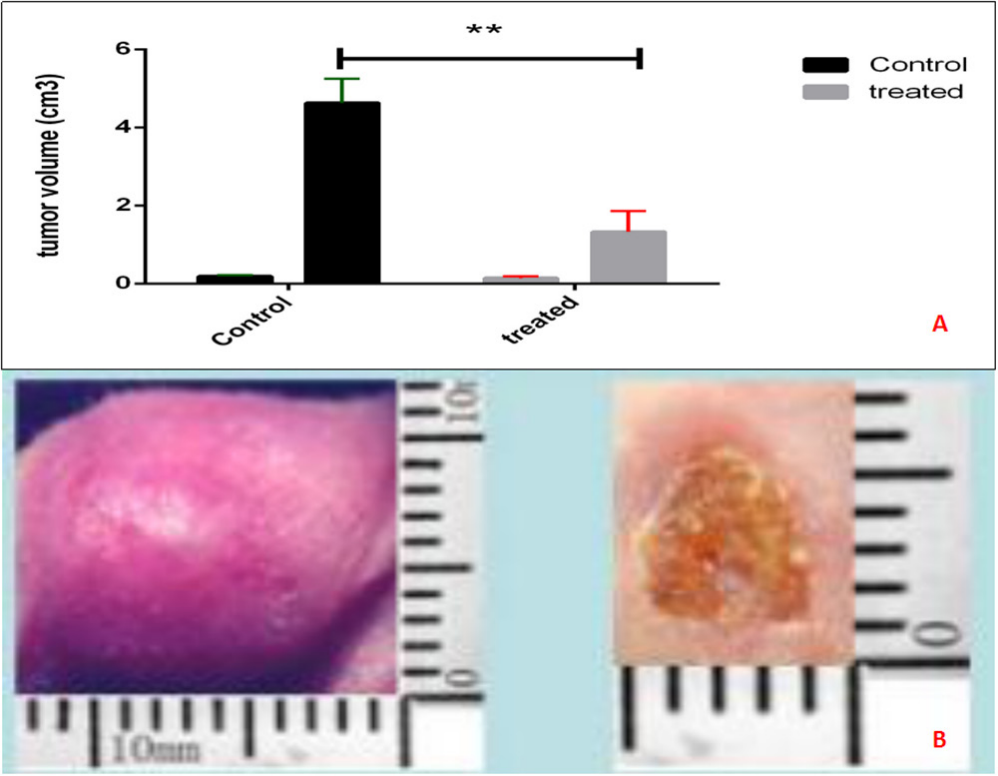
Tumor volume in murine transplanted hepatocellular carcinoma with high metastatic potential After different treatments, the mice were followed up for 14 days. The nsPEF ablation inhibit the tumor vs control group (P< 0.05).

Statistical analysis showed nsPEF ablation significantly reduced the primary tumor volume compared with control group (P <0.05) (Figure 3 and Figure 4).

### NsPEF inhibit serum alkaline phosphatase in dogs

Alkaline phosphatase increases in osteosarcoma. Its level in serum was used to indicate the tumor metabolic activity. In healthy individuals, alkaline phosphatase derives mostly from bone, hepatic tissues, or kidney. It is known that patients with osteosarcoma are commonly detected with increased ALP levels. The relationship between total alkaline phosphatase activity and clinical outcome of osteosarcoma patients has been recognized for over 50 years [11]. Data show serum alkaline phosphatase levels in tumor-bearing control dogs increased when there is no treatment. The surgery removed the tumor and the alkaline phosphatase decreased (p<0.001). NsPEF inhibit alkaline phosphatase significantly (P<0.05 vs control) (Figure 3).

### Tissue damage caused by needle puncture on knee joint capsule

**Figure 5:**
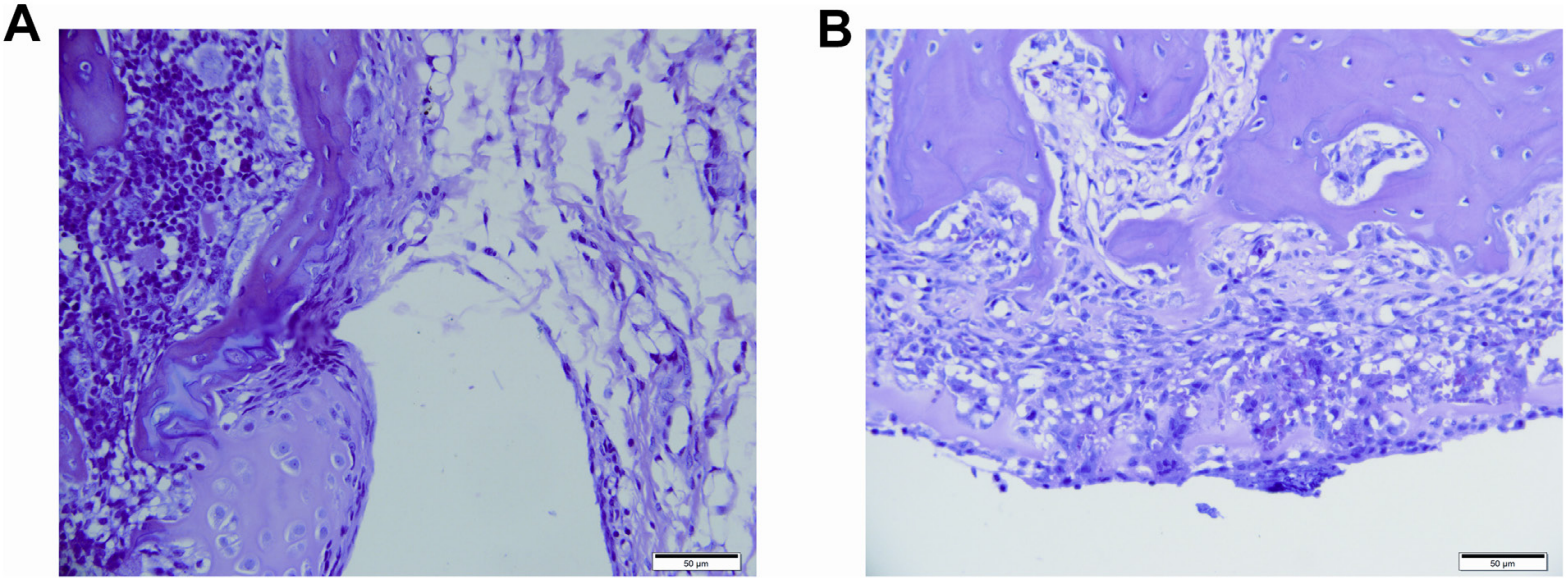
Pathology of dog joint capsule The dog joint capsule was stained by H&E. The puncture electrode pierced the capsule and caused infection after ablation **(Figure 5A)**. Four weeks post treatment, the puncture site showed no infection or joint deformity **(Figure 5B)**. Scale bar 50μm.

The puncture electrode pierced the joint capsule and caused infection (Figure 5A). After four weeks post treatment, the puncture site showed the infection was cleared and there was no joint deformity or capsule destruction (Figure 5B).

### NsPEF inhibits lung metastasis *in vivo*

**Figure 6:**
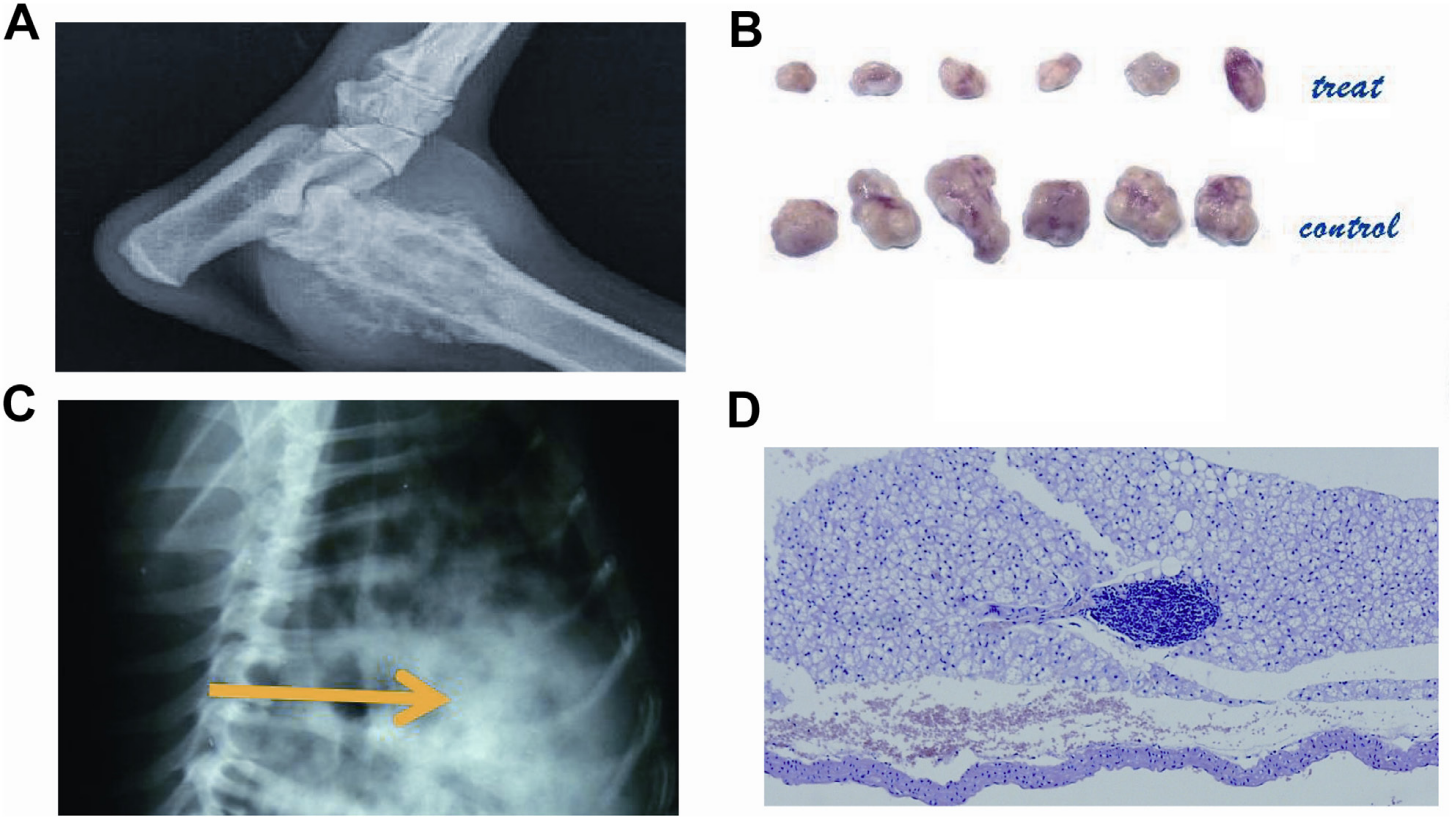
The radiology and pathology of the primary tumor and lung metastasis The primary tumor of canine osteosarcoma **(Figure 6A)**. The primary tumor of murine HCC **(Figure 6B)**. Canine spontaneous osteosarcoma spread to the lung as multiple rounded nodules **(Figure 6C)**. After ablation, the lung metastasis clusters were inhibited as a focal diameter smaller than 1 cm **(Figure 6D)**. (Scale bar 500μm).

The total number of lung metastase nodules found in X-ray film in dogs and pathology in mice were summarized in Table 3. NsPEF ablation and surgery had a significantly lower lung metastasis rate than control group ( P=0.017 in dogs, nsPEF vs control; P=0.014 in mice, nsPEF vs control; P=0.004 in dogs, surgery vs control). (Table 3). The pathology showed metastatic clusters in the control group (Figure 6). After treatment the metastatic tumor nodules decreased in number and became micro focals in the alveolar tissue (Figure 6).

**Table 3:** Summary of the lung metastasis after treatment

	canine spontaneous osteosarcoma			murine transplanted HCC	
	nsPEF(n=4)	control(n=5)	amputation(n=3)	nsPEF(n=6)	control(n=6)
**metastasis number**	1	7	0	3	9
**metastasis rate (%)**	25%(1/4)	100%(5/5)	0(0/3)	33.3%(2/6)	100%(6/6)
***X*^2^**	5.625		8.000	6.000	
***P***	0.017		0.004	0.014	

## DISCUSSION

Novel therapeutic intervention of nsPEF ablation was applied on different tumor bearing animal models with lung metastasis, demonstrating anti-cancer activity in remote metastasis when ablating the primary tumor.

In clinical practice, many patients have had micro-metastasis on diagnosis but the radiological exams cannot detect remote metastasis. Or, the late staging of tumor constrained patients for radical surgery. In these cases the new down-stage ablation and immune stimulation approach are in great need. According to our results, local application of nsPEF decreased ALP. The primary tumor growths were inhibited. The nsPEF showed a survival benefit compared to controls. Local ablation can work as an important part in the systematic tumor control strategy with advantage of a longer survival and less lung metastasis.

The results also showed that nsPEF caused no adverse heart events or major complications during treatment. During ablation the temperature did not exceed 40oC. So it avoids the risk of thermal injuries to adjacent structures as seen in other heat or freezing based ablation methods. The adverse event is needle tract bleeding. When delivering electric energy by a puncture needle, nsPEF causes a temporary injury along the needle track. But follow-up study showed that the joint capsule recovered without deformity.

The minimally invasive nsPEF extend survival and improve quality of life. It offers a nonsurgical, localized treatment that ablates the primary tumor with pulsed power and inhibit the lung metastasis. After nsPEF ablation the legs can resume the usual activities. NsPEF ablation is a minimally invasive treatment for local tumor control with negligible mortality, short hospital stay and positive gain in quality of life. Thus, nsPEF ablation can be used as an alternative treatment for those who are in late stage and contraindicated for surgery with metastatic diseases.

In summary, nsPEF inhibits primary tumor activity and growth; prolongs survival time with minimum invasive ablation procedure, suggesting its anti-cancer benefit in the selected candidates. As a non-thermal ablation method, it has no joint capsule damage. Local nsPEF ablation has advantages in reducing lung metastasis, lower morbidity/infection, shorter hospitalization and relatively lower cost. NsPEF has potential to treat late-stage tumor as a palliative therapy, to improve survival for patients with lung metastasis.

## MATERIALS AND METHODS

### Ethical statement

The experimental protocols were approved by Institutional Animal Care and Use Committee of the First Affiliated Hospital of Zhejiang University. The informed consent was obtained in advance. All methods were performed in accordance with the relevant guidelines and regulations of The Declaration of Helsinki and National Institutes of Health Guide for Care and Use of Laboratory Animals.

### The nsPEF pulse generator

The nsPEF generator was previously described [1–3]. Electric fields released to the osteosarcoma with a self-designed needle puncture electrode (Figure 1) by an oscilloscope (DPO4054, Tektronix, USA). Tumors were exposed to a single nsPEF ablation with optimized parameters (100 ns, 40Kv/cm, 500 pulses, 1 Hz).

### Electrode

Puncture electrode was made by the medullo-puncture needle (pair electrode, 15 mm puncture length, upper holder is protected by insulation) and then sterilized. It can pierce bone and allow smooth puncture performance with the sharp needle tip. The distance in between the needles was 2cm. The electric field distribution was not uniform and formed an oval ablation zone. The machine generated the highest 40 kV/cm-electric field in the center. The tumor temperature during treatment was lower than 40°C. (Figure 1).

### Animals

Animals included in the study were 12 dogs with spontaneous osteosarcoma and 12 mice transplanted with human high metastatic hepatocellular carcinoma. The tumors were classified in late stage by TNM system (Table 1 and Table 2). Animal study was approved by the Institutional Animal Care and Use Committee of the First Affiliated Hospital of Zhejiang University. The informed consent was signed by owners. The study was conducted by a licensed orthopedic surgeon and supervised by certificated veterinarian.

### Study design

Total of 12 dogs with spontaneous osteosarcoma were assigned randomly into 3 groups: nsPEF treatment group (n=4), control group without any treatment (n=5) and amputation surgery group (n=3). Assessment of clinical tumor response was evaluated with 6-month routine follow-up. Tumor samples and blood were collected.

Total of 12BALB/c nude mice were transplanted with high metastatic HCC cell line HCCLM3 according to our previous work [3]. This HCC tumor model has been approved for producing high lung metastasis occurrence [8–10]. They were assigned randomly into 2 groups: nsPEF treatment group (n=6), control group without any treatment (n=6). Assessment of clinical tumor response was evaluated with 14-day routine follow-up. Tumor samples were collected and measured (Figure 6).

### Follow-up

For dogs, blood samples were collected twice (before nsPEF treatment and 6 month post nsPEF). Tumor responses to different treatments were assessed by radiography evaluation of the tumor size. Animals were X-rayed twice (before nsPEF treatment and 6-month post nsPEF). The numbers of lung metastasis nodules were counted on X-ray films. The animals were schedule to be euthanized after 6 months. But whenever the limb tumor grew to be larger than 5 cm or lung metastasis caused severe breathing difficulty the animal was then euthanized. Observation and sample collection took place at the time of euthanisia. For mice, HCC tumors were measured and assessed by gross anatomy (Figure 6). The numbers of lung metastasis nodules were checked on lung pathological slides. The mice were euthanized when tumors exceed 2cm.

### Statistical analysis

Results were displayed as mean ± standard deviations (SD) and then analyzed by ANOVA (SPSS V17.0, Chicago, IL, USA). Survival times were present by Kaplan-Meier survival curve. The lung metastasis occurrence rate between treatment and control group was compared by chi-square test. Statistical significance was determined by using P < 0.05 as a significant level.

## Abbreviations

nsPEF: nanosecond pulsed electric field
ALP: alkaline phosphatase
NPS: nano-pulse stimulation

## References

[1] Ren Z, Chen X, Cui G, Yin S, Chen L, Jiang J, Hu Z, Xie H, Zheng S, Zhou L (2013). Nanosecond pulsed electric field inhibits cancer growth followed by alteration in expressions of NF-κB and Wnt/β-catenin signaling molecules. PLoS One.

[2] Chen X, Yin S, Hu C, Chen X, Jiang K, Ye S, Feng X, Fan S, Xie H, Zhou L, Zheng S (2014). Comparative study of nanosecond electric fields in vitro and in vivo on hepatocellular carcinoma indicate macrophage infiltration contribute to tumor ablation in vivo. PLoS One.

[3] Yin S, Chen X, Hu C, Zhang X, Hu Z, Yu J, Feng X, Jiang K, Ye S, Shen K, Xie H, Zhou L, R James Swanson (2014). Nanosecond pulsed electric field (nsPEF) treatment for hepatocellular carcinoma: a novel locoregional ablation decreasing lung metastasis. Cancer Lett.

[4] Chen R, Sain NM, Harlow KT, Chen YJ, Shires PK, Heller R, Beebe SJ (2014). A protective effect after clearance of orthotopic rat hepatocellular carcinoma by nanosecond pulsed electric fields. Eur J Cancer.

[5] Nuccitelli R, Berridge JC, Mallon Z, Kreis M, Athos B, Nuccitelli P (2015). Nanoelectroablation of Murine Tumors Triggers a CD8-Dependent Inhibition of Secondary Tumor Growth. PLoS One.

[6] Pliquett U, Nuccitelli R (2014). Measurement and simulation of Joule heating during treatment of B-16 melanoma tumors in mice with nanosecond pulsed electric fields. Bioelectrochemistry.

[7] Nuccitelli R Optimizing Nano-Pulse Electro-Signalling (NPES) Parameters for Activating Immunogenic Apoptosis and Inhibiting Metastasis. http://www.bioelectrics2016.org.

[8] Li Y, Tang Y, Ye L, Liu B, Liu K, Chen J, Xue Q (2003). Establishment of a hepatocellular carcinoma cell line with unique metastatic characteristics through in vivo selection and screening for metastasis-related genes through cDNA microarray. J Cancer Res Clin Oncol.

[9] Yang BW, Liang Y, Xia JL, Sun HC, Wang L, Zhang JB, Tang ZY, Liu KD, Chen J, Xue Q, Chen J, Gao DM, Wu WZ (2008). Biological characteristics of fluorescent protein-expressing human hepatocellular carcinoma xenograft model in nude mice. Eur J Gastroenterol Hepatol.

[10] Zhang ZJ, Yang YK, Wu WZ (2014). Bufalin attenuates the stage and metastatic potential of hepatocellular carcinoma in nude mice. J Transl Med.

[11] Ren HY, Sun LL, Li HY, Ye ZM (2015). Prognostic Significance of Serum Alkaline Phosphatase Level in Osteosarcoma: A Meta-Analysis of Published Data. Biomed Res Int.

